# *Schizophyllum commune*-associated allergic bronchopulmonary mycosis in a postoperative lung cancer patient: A case report and comprehensive literature review

**DOI:** 10.1097/MD.0000000000047480

**Published:** 2026-02-13

**Authors:** Ningpei Wu, Yi Liang, Qunfeng Yan

**Affiliations:** a Department of Pulmonary and Critical Care Medicine, Ninghai First Hospital, Ningbo City, Zhejiang, China; b Department of Pulmonary and Critical Care Medicine, Medical School, Ningbo University, Ningbo City, Zhejiang, China.

**Keywords:** ABPM, lung cancer, *S commune*, sinonasal mycosis

## Abstract

**Rationale:**

*Schizophyllum commune (S commune*), a basidiomycetous fungus, is increasingly recognized as an important cause of allergic bronchopulmonary mycosis, particularly in immunocompetent individuals with a history of respiratory surgery, chronic sinusitis, or structural airway abnormalities.

**Patient concerns:**

A 71-year-old female with a prior right upper lobectomy for lung cancer and a history of invasive fungal sinusitis presented with recurrent dyspnea, cough, and chest tightness.

**Diagnoses:**

The diagnosis was confirmed by fungal culture and 18S rRNA sequencing, along with significantly elevated total serum immunoglobulin E (541 IU/mL), specific immunoglobulin E to *S commune* (15.2 kU/L), and peripheral eosinophilia (8.0%).

**Interventions:**

The patient was treated with voriconazole and inhaled corticosteroids/formoterol for asthma control.

**Outcomes:**

Marked improvement in symptoms and radiologic findings was observed after 1 month of treatment, with sustained improvement over 3 months.

**Lessons:**

This case highlights the diagnostic challenges in distinguishing *S commune*–induced allergic bronchopulmonary mycosis and emphasizes the importance of considering this pathogen even in immunocompetent patients.

## 1. Introduction

Allergic bronchopulmonary mycosis (ABPM) represents an allergic airway disorder induced by type I and type III hypersensitivity reactions to environmental fungi, characterized by mucus plugs, bronchiectasis, and eosinophilic inflammation. Unlike allergic bronchopulmonary aspergillosis, which is predominantly caused by *Aspergillus fumigatus*, ABPM encompasses a wider range of fungal pathogens. *S commune*, a basidiomycetous fungus, has emerged as one of the most common non-Aspergillus causes of ABPM, particularly in Japan, where it accounts for a significant proportion of reported cases. We report a rare case of *S commune*-induced ABPM in a postoperative lung cancer patient and review the relevant published literature worldwide to provide insights into its clinical presentation, diagnosis, and management (Table 1). To identify relevant case reports on ABPM caused by *S commune*, we conducted a comprehensive literature review using PubMed, searching for articles published from 2007 to the present. The search terms used were “*S commune*” and “ABPM”.

**Table 1:** Case reports summary on allergic bronchopulmonary mycosis due to *Schizophyllum commune*.

#	Yr	Nation	Sex	Age	History	Symptom	Physical exam	Lab exam	Image (CT)	Site	Bronchoscopy	Treatment	Duration	Prognosis
1^[1]^	2009	Japan	M	55	Asthma; history of pneumonia (2003, 2005)	Fever, cough, sputum	Normal respiratory sounds; no wheezing	WBC: 10,400/µL; eosinophils: 15.9; total IgE: 3592 IU/mL; positive for Aspergillus IgE	Infiltrates and mucoid impaction	Left upper lobe, right lower lobe	Mucous plug with Charcot–Leyden crystals & fungal hyphae	Fungal hyphae;	Itraconazole (400 mg/d) and corticosteroid (prednisolone, 30 mg/d)	4 mo, followed by maintenance therapy, but recurrence after 1 yr
2^[2]^	2014	Japan	F	61	Bronchial asthma and eosinophilic pneumonia for 5 yr	Chronic cough, increased sputum, dyspnea, wheezing	Bilateral coarse crackles with biphasic polyphonic wheezes	Eosinophils: 861/µL (6–20); total IgE: normal (70.36 IU/mL); positive Aspergillus-specific IgE	Bilateral bronchiectasis, hyperattenuating mucus in the right upper lobe (CT)	Bilateral central bronchi	DNA analysis confirmed *Aspergillus fumigatus* and *Schizophyllum commune*	Oral itraconazole and prednisolone	Not specified	Improvement in symptoms and imaging
3^[3]^	2019	Japan	M	42	Asthma	Chronic cough, sputum, dyspnea	Bilateral rhonchi and wheezes	Eosinophils: 670/µL; total IgE: normal (42 IU/mL); initial IgE and IgG for *Schizophyllum commune* were negative, IgG positive after 7 yr	Bronchiectasis with mucus impaction in the right upper lobe (CT)	Right upper and left lower lobes	Mucus plugs cultured *Schizophyllum commune*	Initial: ICS + LABA; relapse: itraconazole (200 mg/d) and prednisolone (40 mg/d)	4 yr follow-up	Improvement observed, no relapse over 4 yr
4^[4]^	2023	Japan	M	61	Childhood asthma	Dry cough for 1 mo, abnormal chest radiograph	Normal breath sounds	Eosinophils: 945 cells/µL; total IgE: 199 U/mL; positive for *Schizophyllum commune*-specific IgE and IgG, negative for Aspergillus-specific IgE/IgG	Bronchiectasis with high-attenuation mucus and consolidation in right upper lobe (S3 segment, CT)	Right upper lobe (S3)	Bronchoscopy revealed yellowish-white mucus plug; cultured *Schizophyllum commune*	Inhaled corticosteroid/long-acting β2-agonist (fluticasone furoate/vilanterol)	3 mo	Improvement observed; mucus plugs resolved on CT, no relapse at 8-mo follow-up
5^[5]^	2010	Japan	F	71	Long-term history of asthma	Chest discomfort, dyspnea, cough, wheezing	Wheezes and rhonchi on auscultation	Eosinophils: 0.7; total IgE: 35 U/mL; negative for *Schizophyllum commune*-specific IgE and IgG	Normal chest and sinus CT findings	No bronchiectasis or mucus plugs	Positive bronchial provocation test for *Schizophyllum commune*	Inhaled corticosteroids, montelukast, theophylline, budesonide/formoterol, tiotropium, itraconazole (50 mg/d for 2 wk)	2 wk	Improvement in symptoms; ACT score increased from 13 to 16
6^[5]^	2010	Japan	M	69	Asthma history (5 yr)	Asthma attacks, cough, shortness of breath	Late expiratory wheezes	Eosinophils: 2.0; total IgE: 114 U/mL; positive for *Schizophyllum commune*-specific IgE and IgG	Normal chest and sinus CT findings	No bronchiectasis or mucus plugs	Positive bronchial provocation test for *Schizophyllum commune*	Inhaled corticosteroids, montelukast, theophylline, itraconazole (50 mg/d for 2 wk)	2 wk	Improvement observed; decrease in specific IgG levels
7^[6]^	2011	India	F	35	Long-term exposure to passive smoke and biomass fuel; history of chronic suppurative otitis media and treated pulmonary tuberculosis	Cough with purulent sputum, dyspnea, wheezing	Bilateral coarse crepitations with biphasic polyphonic rhonchi	Eosinophils: 980 cells/µL; total IgE: 2448 IU/mL; elevated specific IgE for *Schizophyllum commune*, negative for Aspergillus spp.	Bilateral central bronchiectasis, high-attenuation mucus in the left bronchi (CT)	Bilateral central bronchi	Bronchial secretions cultured *Schizophyllum commune*	Oral prednisolone and inhaled corticosteroids	Not specified	Symptomatic improvement
8^[6]^	2011	India	M	42	Diabetes mellitus for 10 yr, previously treated for pulmonary tuberculosis	Occasional hemoptysis	Pallor	Elevated total IgE and specific IgE for *Schizophyllum commune*	Air-crescent sign in the right upper lobe (CT), indicating fungal ball	Right upper lobe	Sputum culture confirmed *Schizophyllum commune*	Oral prednisolone and itraconazole (200 mg twice daily)	4 mo	Symptomatic improvement
9^[7]^	2023	China	F	49	Hepatitis B (under oral entecavir treatment)	Recurrent chronic cough	No abnormalities on auscultation	Eosinophils: 0.85 × 10^9^/L; total IgE: 1280 IU/mL	Hyperattenuated mucoid impaction	Right middle lobe	Yellow mucous plugs obstructing right middle lobe	Voriconazole (200 mg twice daily) and prednisolone (20 mg daily)	3 mo	No relapse observed at 6-mo follow-up
10^[8]^	2024	China	F	49	Chronic hepatitis B	Chronic cough, sputum, and dyspnea for 6 mo	Decreased breath sounds on the right side, no rales or wheezes	Eosinophils: 0.85 × 10^9^/L; total IgE: 1280 IU/mL; elevated HBV DNA: 2.16 × 10^6^ IU/mL	Mucus plugs in the right middle lobe, bronchiectasis, central lung atelectasis (CT)	Right middle lobe	Bronchoscopy revealed white mucus plugs.	Voriconazole (400 mg/d) and prednisone (20 mg/d) combined with entecavir for hepatitis B	6 mo	Improvement observed, no relapse at 6-mo follow-up
11^[9]^	2007	Japan	F	75	Chronic hepatitis C	Productive cough	Normal breath sounds with occasional coarse crackles	Eosinophils: 4.9; total IgE: 6448 IU/mL; Aspergillus antibody negative	Right lower lobe atelectasis with central bronchiectasis and centrilobular nodules (CT)	Right lower lobe	Bronchoscopy revealed abundant mucus plugs; cultured *Schizophyllum commune*	Itraconazole 400 mg/d; discontinued due to heart failure, readministered at 100 mg/d upon recurrence	Initial treatment for 3 mo, re-treatment upon recurrence	Improved; no recurrence 3 mo after discontinuation
12^[10]^	2021	Japan	M	76	Diabetes mellitus, hypertension	Hemoptysis for 19 mo	Temperature: 36.2°C, heart rate: 76 bpm, oxygen saturation: 97	Eosinophils: 177/µL; Aspergillus antibody negative	Cavity with internal nodule (42 × 24 mm, 13 × 11 mm) in the left upper lobe (CT)	Left upper lobe	Confirmed *Schizophyllum commune* via ITS sequencing; initial bronchoscopy was inconclusive	Left upper lobectomy	5 mo	Improvement observed, no recurrence at 5-mo follow-up
13^[11]^	2022	Japan	F	78	No history of asthma or smoking	Prolonged cough	Normal physical examination	Normal IgE; elevated CEA: 15.6 ng/mL, CYFRA 21-1: 3.6 ng/mL	Left lower lobe mass, right middle lobe collapse (CT)	Left lower lobe, right middle lobe	Bronchoscopy showed mucus plugs, eosinophils, Charcot–Leyden crystals, and fungal mycelia detected	Corticosteroid therapy initiated	10 mo	Improvement observed, no relapse after 5 mo
14^[12]^	2024	Japan	F	63	No history of bronchial asthma	Wet cough for 3 mo, chest pain for 3 d	No abnormal lung sounds	Eosinophils: 688/µL; total IgE: 1522 IU/mL; positive *Aspergillus fumigatus* IgE (2.24 UA/mL), Asp f 1-specific IgE negative	High-attenuation mucus in the right middle lobe (CT), yellow mucus plug in bronchi	Right middle lobe	A yellow mucus plug in the right B5 bronchus	Prednisolone (0.5 mg/kg), followed by itraconazole (200 mg/d)	3 mo	Improvement observed, no relapse at 104-d follow-up
15^[13]^	1994	Japan	F	57	No history of asthma; history of recurrent colds and sinusitis	Mild cough with occasional sputum	No abnormalities on physical examination	Eosinophils: 12; total IgE: 4286 U/mL; positive IgG for *Schizophyllum commune* (1:320)	Infiltrates and nodules in the left lingular lobe and right upper lobe (CT)	Left lingular and right upper lobes	Bronchoscopic analysis identified *Schizophyllum commune*, initially misidentified as Aspergillus	No antifungal or steroid treatment; spontaneous symptom remission	Not specified	Symptoms fluctuated, but no significant deterioration
16^[14]^	2007	Japan	F	58	No prior history of asthma	Cough, sputum, pulmonary infiltrates	Normal physical examination	Eosinophils: 20.9; total IgE: 805 U/mL; positive for *Schizophyllum commune*-specific IgE and IgG	High-attenuation mucus in left B8 bronchus; bronchiectasis (CT)	Left lower lobe	Bronchoscopy revealed green mucus plugs; *Schizophyllum commune* isolated	Bronchial clearance	Not specified	Symptom relief; no steroids used; no recurrence over 4 yr
17^[14]^	2009	Japan	F	70	No prior history of asthma	Cough, high fever, general malaise	Temperature: 38°C; heart rate: 100 bpm	Eosinophils: 9.4; total IgE: 787 U/mL; positive for *Schizophyllum commune*-specific IgE and IgG	Mucoid impaction in right upper lobe (S3) (CT)	Right upper lobe	Bronchial washings confirmed *Schizophyllum commune* and *Candida albicans*	Low-dose itraconazole and bronchial clearance	3 mo	Improvement observed; no relapse within 6 mo
18^[15]^	2007	Japan	F	64	No known asthma history	Cough, hemoptysis, right anterior chest pain	Normal breath sounds, no wheezes	Eosinophils: 15 (1072/µL); total IgE: 1340 IU/mL; Aspergillus antibody negative	Hyperattenuating mucoid impaction in right upper lobe (S3b) with minor pleural effusion (CT)	Right upper lobe	Bronchial lavage culture confirmed *Schizophyllum commune*	Itraconazole (200 mg/d) and prednisolone (15 mg/d); followed by inhaled steroids (fluticasone)	1 yr	Improvement in symptoms and imaging; no recurrence during follow-up
19^[16]^	2018	Japan	F	63	No history of smoking or dust exposure	Cough, sputum, abnormal chest X-ray shadows	No significant asthma symptoms	Eosinophils: 300/µL; total IgE: 1363 IU/mL (increased to 1439 IU/mL, decreased to 547 IU/mL)	Mucoid impaction in the left middle lung field; lingular bronchus involvement (CT)	Lingular bronchus	Mucous plug culture confirmed *Schizophyllum commune*	Initial: itraconazole (200 mg/d, 16 wk) ineffective; follow-up: voriconazole (1 yr)	1 yr	Improvement; mucus plugs cleared on CT; no relapse after 2 yr

CT = computed tomography, F = female, M=male, IgE = immunoglobulin E.

## 2. Case presentation

A 71-year-old woman with a history of right upper lobectomy for lung cancer was admitted to our hospital due to recurrent chest tightness, dyspnea, and cough with sputum over the past 1.5 years. The patient underwent right upper lobectomy under general anesthesia in November 2018. The postoperative pathology indicated lung adenocarcinoma, staged as T1bN0M0, IA stage. She did not receive adjuvant chemotherapy or radiation therapy postoperatively. Eight months later, she presented with symptoms and findings suggestive of invasive fungal sinusitis, leading to the decision for functional endoscopic sinus surgery. However, due to the lack of microbiological confirmation, a definitive diagnosis of invasive fungal sinusitis could not be established, and therefore, no antifungal treatment was initiated post-surgery. The specific fungal pathogen remained unidentified at that time.

Approximately 1 year after the lung cancer surgery, the patient began to experience asthma symptoms, characterized by dyspnea on exertion and nocturnal cough. At that time, her primary physician conducted pulmonary function tests, which revealed a decreased forced expiratory volume in 1 second (FEV1) to forced vital capacity ratio of 0.65, indicating obstructive ventilatory dysfunction. Specifically, her FEV1 was measured at 1.52 L. To manage these symptoms, she intermittently used inhaled corticosteroids (such as budesonide) and long-acting bronchodilators (such as formoterol) prescribed by her primary physician. However, her symptoms remained poorly controlled prior to the diagnosis of fungal infection.

**Figure 1.**
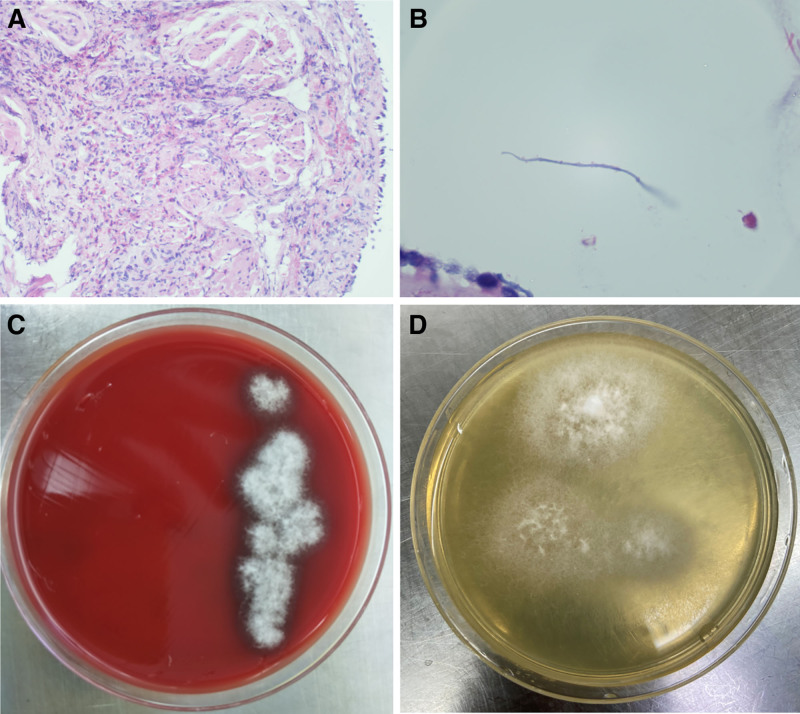
(A) Large amounts of eosinophils were seen in the biopsied tissue. (B) Hyphae with rope-like and small spine-like structures under the microscope. (C–D) White, woolly colonies of *S commune* in Sabouraud blood and dextrose agar after incubation.

In March 2020, the patient underwent a chest computed tomography (CT) scan due to recurrent symptoms of chest tightness, dyspnea, and cough. The CT revealed patchy shadows in the right lower lobe. To identify the pathogen, sputum culture was performed, which yielded negative results. Based on the clinical presentation and imaging findings, she was treated with empirical antibiotics, specifically Moxifloxacin. Her symptoms subsequently improved. However, 5 months later, she experienced a recurrence of chest tightness and dyspnea, which were more severe than before. A follow-up chest CT 6 months later showed a high-density cast-like lesion in the right lower lobe, which had increased in size compared to the previous scan. This raised concerns for either tumor recurrence or fungal infection (Fig. 1A).

**Figure 2.**
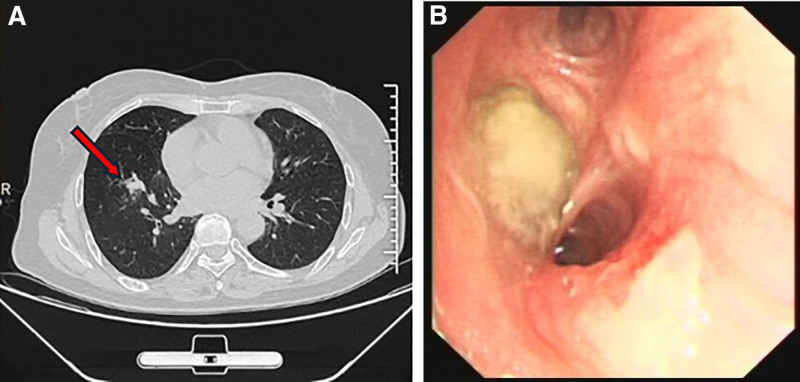
(A) CT scan revealed a cast-like high-density shadow in the right lower lobe of the lung. (B) Bronchoscopy revealed mucosal swelling and thick white mucous plugs in the right lower bronchus, which could be partially aspirated. CT = computed tomography.

Upon admission, a comprehensive physical examination was conducted, which included both general and chest-specific assessments. No abnormal signs were detected during this thorough evaluation. Laboratory tests showed a white blood cell count of 5.84 × 10^9^/L, with 0.47 × 10^9^/L eosinophils (8.0% of total white blood cells). Blood tests also revealed elevated total serum immunoglobulin E (IgE) levels at 541 IU/mL and C-reactive protein at 0.79 mg/L. Chest CT indicated increased density in the right lower lobe, suggesting possible fungal involvement. Bronchoscopy was then performed, revealing mucosal swelling and thick white mucous plugs in the right lower bronchus, which could be partially aspirated (Fig. 1B). Analysis of bronchoalveolar lavage fluid showed 10% eosinophils, and fungal culture confirmed the presence of *S commune*. The differential diagnosis included ABPM and other fungal infections. The culture and sequencing of bronchoalveolar lavage fluid and bronchial brush samples identified *S commune*, a filamentous fungus, through 18S rRNA sequencing (Fig. 2).

To further support the diagnosis of ABPM, specific IgE and skin tests were conducted. The specific IgE test revealed significantly elevated levels of specific IgE against *S commune* (15.2 kU/L, with a normal value of <0.35 kU/L), indicating an allergic reaction to *S commune*. Additionally, the skin test also yielded a positive response, further confirming the patient’s allergic state to *S commune*. These test results provided strong evidence for the diagnosis of ABPM.

After the diagnosis of ABPM, the patient was initiated on a treatment regimen of voriconazole and inhaled corticosteroids/formoterol for asthma control. One month into the treatment, her symptoms significantly improved, with decreased peripheral eosinophil levels and IgE levels. A repeat chest CT showed remarkable resolution of the lung lesion. She continued the same treatment for 3 months, during which no side effects or recurrence were observed. During the 3-month follow-up, the patient reported sustained improvement in symptoms. She experienced reduced frequency and severity of chest tightness and dyspnea, and her cough had also diminished. In the third month of treatment, we conducted a follow-up that included pulmonary function tests and chest CT. Pulmonary function tests indicated improved lung function, particularly an increased ratio of FEV1 to forced vital capacity, which rose from 0.65 before treatment to 0.74 after treatment. Specifically, the patient’s FEV1 increased from 1.52 L before treatment to 1.70 L after treatment. Chest CT revealed near-complete absorption of the lesion in the right lower lobe, with no new lesions detected. The patient expressed satisfaction with the treatment outcome and committed to continuing the prescribed treatment and regular follow-ups as advised.

## 3. Discussion

*S commune*, a basidiomycetous fungus, is increasingly recognized as a cause of ABPM, particularly in Japan.^[17]^ ABPM caused by *S commune* is more commonly associated with central bronchiectasis and mucus plugs compared to the more prevalent ABPA.^[18]^ This fungus thrives in warm and humid environments, such as the human airway, making it highly suited for colonization under favorable conditions.^[19]^ Structural changes in the airway and potential impairment of mucociliary clearance, such as those observed in postoperative lung cancer patients, may create a niche conducive to fungal persistence and allergic sensitization, raising the question of whether surgical interventions increase susceptibility to ABPM caused by *S commune*. This case highlights the clinical significance of ABPM caused by *S commune* in the context of postoperative lung cancer, where fungal colonization and allergic responses may exacerbate pulmonary symptoms.

The diagnostic criteria for ABPM by Asano et al include asthma, eosinophilia, elevated IgE, positive skin test, central bronchiectasis, positive fungal culture, hyphae in mucus plugs, positive serology, and bronchial mucus plugs.^[20]^ Our patient meets criteria 1 (asthma), 2 (eosinophilia), 3 (elevated IgE), 6 (fungal culture), 7 (hyphae in mucus plugs), and 9 (bronchial mucus plugs), confirming ABPM caused by *S commune*. With the advancement of diagnostic technologies, including next-generation sequencing and fungal culture methods, diagnosing *S commune*-related ABPM has become increasingly straightforward. An analysis of 19 reported cases of *S commune*-related ABPM revealed distinct clinical and demographic characteristics, with the majority of patients being middle-aged to elderly women. Many cases had predisposing conditions such as asthma^[1–5]^ or pulmonary tuberculosis,^[6]^ underscoring the pivotal role of underlying pulmonary diseases and structural airway changes in disease progression. Additionally, several cases were linked to immunocompromised states, including chronic hepatitis B^[7–9]^ and diabetes mellitus,^[10]^ further emphasizing the contribution of systemic immune dysregulation to fungal colonization and hypersensitivity (Table 1).

Structural changes in the airway, such as those observed in postoperative lung cancer patients, may play a pivotal role in facilitating fungal colonization and hypersensitivity reactions. *S commune*, an environmental fungus, thrives in disrupted anatomical environments, where impaired mucociliary clearance and localized immune dysregulation create a niche for its persistence. In one reported case, a 76-year-old man with diabetes mellitus and hypertension was diagnosed with a fungal ball caused by *S commune* within a squamous cell carcinoma cavity, highlighting the interplay between tumor-associated structural changes and fungal colonization.^[10]^ Similarly, our case underscores the significance of structural airway changes in the development of *S commune*-related ABPM. The patient’s history of right upper lobectomy for lung cancer and prior sinus surgery for invasive fungal infection likely contributed to an environment conducive to fungal persistence and allergic sensitization. The absence of antifungal therapy following sinus surgery may have allowed residual fungal elements to act as a source for reinfection or sensitization in the lower airways.

Distinguishing *S commune*-related ABPM from lung cancer presents a significant diagnostic challenge, particularly in high-risk patients with structural airway changes or immune modulation. Imaging studies in such patients often reveal findings that mimic malignancy, such as pulmonary lesions, high-attenuation mucus, or even increased fluorodeoxyglucose uptake on PET-CT. For example, a reported case described a patient with elevated serum carcinoembryonic antigen levels and fluorodeoxyglucose-avid pulmonary shadows, initially raising concerns for lung cancer recurrence.^[11]^ However, subsequent bronchoscopy revealed fungal colonization and mucus plugs, confirming the diagnosis of ABPM. Similarly, our patient demonstrated pulmonary imaging abnormalities and a history of lung cancer, which initially raised concerns for tumor recurrence. Definitive evidence of ABPM was established through findings of eosinophilia, elevated.

IgE levels and fungal identification via bronchoscopy were assessed. These cases highlight the overlap in imaging and laboratory findings between fungal infections and malignancies, the importance of integrating clinical, laboratory, and advanced diagnostic tools, such as next-generation sequencing, to ensure accurate differentiation. Given *S commune*’s ability to adapt to structural environments, such as surgical alterations or cancerous cavities, ABPM should always be considered in patients with eosinophilia, high-attenuation mucus, or bronchiectasis, particularly when structural airway changes are evident. Early recognition and precise diagnosis are critical to providing appropriate treatment, avoiding unnecessary interventions, and improving outcomes in this vulnerable population.

On CT scans, *S commune*-related ABPM primarily manifests as mucoid impaction, which is commonly found in the right middle lobe, left upper lobe, and right upper lobe.^[1–3,7–9,12,13,18,20]^ This condition often coexists with bronchiectasis, presenting as dilation of the bronchial lumen in the bilateral central bronchi or specific lobes.^[1–3,7–9,12–14]^ In addition, pulmonary infiltrates are observed in some cases, while pulmonary nodules appear in a few cases, and pulmonary cavitation is seen in individual cases. Other manifestations include atelectasis and pleural thickening. These CT findings reflect the airway inflammation, increased mucus production, and potential tissue destruction and repair processes resulting from the allergic reaction of the body to fungal infection.

An analysis of 19 reported cases of *S commune*-related ABPM revealed that itraconazole is typically the first-line antifungal agent,^[14,15]^ with voriconazole reserved for cases unresponsive to initial therapy.^[16]^ Corticosteroids, such as prednisone, are frequently coadministered to alleviate airway inflammation. Treatment durations vary, averaging 3 to 4 months, and are adjusted based on clinical and laboratory responses. Most patients experience significant symptom improvement with treatment, though recurrences have been documented. Long-term outcomes are generally favorable.

In this case, voriconazole was chosen as the antifungal agent due to the patient’s postoperative lung cancer status, which involved significant structural airway changes that may reduce the effectiveness of itraconazole. Voriconazole was preferred for its superior tissue penetration and enhanced activity against *S commune*, ensuring adequate antifungal coverage in the altered lung environment. Additionally, the patient’s history of invasive fungal sinusitis raised concerns for potential resistance or partial treatment failure with itraconazole, further supporting the decision to initiate voriconazole. The use of inhaled corticosteroids combined with formoterol further addressed the patient’s concurrent asthma-like symptoms, reducing airway inflammation and improving pulmonary function. Following 1 month of treatment, the patient experienced significant symptomatic relief, with resolution of chest tightness and cough, accompanied by marked reductions in peripheral eosinophil levels and serum IgE. Repeat imaging showed remarkable regression of the pulmonary lesion, confirming the effectiveness of the combined treatment approach.

## Author contributions

**Conceptualization:** Ningpei Wu.

**Data curation:** Ningpei Wu, Yi Liang.

**Formal analysis:** Ningpei Wu.

**Writing – original draft:** Ningpei Wu, Yi Liang.

**Writing – review & editing:** Qunfeng Yan.
